# Unveiling Pharmacogenomics Insights into Circular RNAs: Toward Precision Medicine in Cancer Therapy

**DOI:** 10.3390/biom15040535

**Published:** 2025-04-05

**Authors:** Saud Alqahtani, Taha Alqahtani, Krishnaraju Venkatesan, Durgaramani Sivadasan, Rehab Ahmed, Hassabelrasoul Elfadil, Premalatha Paulsamy, Kalaiselvi Periannan

**Affiliations:** 1 Department of Pharmacology, College of Pharmacy, King Khalid University, Abha 62521, Saudi Arabia; saalqhtany@kku.edu.sa (S.A.); ttaha@kku.edu.sa (T.A.); 2 Department of Pharmaceutics, College of Pharmacy, Jazan University, P.O. Box 114, Jazan 45142, Saudi Arabia; dsivadasa@jazanu.edu.sa; 3 Division of Microbiology, Immunology and Biotechnology, Department of Natural Products and Alternative Medicine, Faculty of Pharmacy, University of Tabuk, Tabuk 71491, Saudi Arabia; rahmed@ut.edu.sa (R.A.); habdelgadir@ut.edu.sa (H.E.); 4 College of Nursing, Mahalah Branch for Girls, King Khalid University, Abha 62521, Saudi Arabia; pponnuthai@kku.edu.sa; 5 Department of Mental Health Nursing, Oxford School of Nursing & Midwifery, Faculty of Health and Life Sciences, Oxford Brookes University, Oxford OX3 0FL, UK; kperiannan@brookes.ac.uk

**Keywords:** pharmacogenomics, circular RNAs, precision medicine, chemotherapy, immunotherapy, targeted therapy, cancer

## Abstract

Pharmacogenomics is revolutionizing precision medicine by enabling tailored therapeutic strategies based on an individual genetic and molecular profile. Circular RNAs (circRNAs), a distinct subclass of endogenous non-coding RNAs, have recently emerged as key regulators of drug resistance, tumor progression, and therapeutic responses. Their covalently closed circular structure provides exceptional stability and resistance to exonuclease degradation, positioning them as reliable biomarkers and novel therapeutic targets in cancer management. This review provides a comprehensive analysis of the interplay between circRNAs and pharmacogenomics, focusing on their role in modulating drug metabolism, therapeutic efficacy, and toxicity profiles. We examine how circRNA-mediated regulatory networks influence chemotherapy resistance, alter targeted therapy responses, and impact immunotherapy outcomes. Additionally, we discuss emerging experimental tools and bioinformatics techniques for studying circRNAs, including multi-omics integration, machine learning-driven biomarker discovery, and high-throughput sequencing technologies. Beyond their diagnostic potential, circRNAs are being actively explored as therapeutic agents and drug delivery vehicles. Recent advancements in circRNA-based vaccines, engineered CAR-T cells, and synthetic circRNA therapeutics highlight their transformative potential in oncology. Furthermore, we address the challenges of standardization, reproducibility, and clinical translation, emphasizing the need for rigorous biomarker validation and regulatory frameworks to facilitate their integration into clinical practice. By incorporating circRNA profiling into pharmacogenomic strategies, this review underscores a paradigm shift toward highly personalized cancer therapies. circRNAs hold immense potential to overcome drug resistance, enhance treatment efficacy, and optimize patient outcomes, marking a significant advancement in precision oncology.

## 1. Introduction

### 1.1. Background on Precision Medicine and Pharmacogenomics

Precision medicine in oncology customizes cancer treatment based on an individual unique genetic, environmental, and lifestyle factors [1]. By leveraging patient-specific tumor profiling, this approach enhances therapeutic efficacy while minimizing adverse effects [2]. Advances in biological databases, genomic sequencing, and computational tools have driven the field forward, enabling the identification of molecular changes that drive cancer progression and facilitating the selection of targeted therapies with improved efficacy and reduced toxicity [1,3,4]. Next-generation sequencing (NGS) has significantly contributed to detailed tumor profiling, allowing for greater precision in treatment selection [5]. Recent breakthroughs in multi-omics integration, such as spatial transcriptomics and proteomics, are refining patient stratification and improving real-time monitoring of treatment responses [6].

Despite its promise, integrating precision oncology into clinical practice faces several challenges. Regulatory and ethical barriers pose significant hurdles, requiring adherence to stringent approval frameworks such as those set by the U.S. Food and Drug Administration (FDA) and the European Medicines Agency (EMA) [7]. Additionally, ethical concerns regarding genomic data privacy, AI-driven patient stratification biases, and informed consent have become central issues in clinical implementation. Ensuring equitable access to precision oncology is also critical, as disparities in genomic data representation may introduce biases in AI-driven pharmacogenomic predictions [8,9]. Economic challenges also hinder accessibility, as the high costs associated with NGS-based tumor profiling, AI-driven drug selection, and targeted therapies limit their widespread clinical adoption, particularly in low-income healthcare systems [7]. Furthermore, specialized training is required for oncologists to interpret multi-omics data and pharmacogenomic reports effectively, highlighting the need for AI-powered decision-support tools to facilitate personalized treatment plans [10]. Addressing these challenges is essential for realizing the full potential of precision medicine in oncology.

### 1.2. Advances in AI-Driven Pharmacogenomics

Precision oncology incorporates diagnostic methodologies, data integration techniques, and therapeutic interventions. Diagnostic strategies, such as liquid biopsy and genomic sequencing, help identify cancer-specific biomarkers and mutations, while AI-driven pharmacogenomics models predict drug response and personalize therapies with improved accuracy [8]. AI plays a crucial role in integrating diverse biological datasets, including genomics, proteomics, and metabolomics, to generate comprehensive tumor profiles, which enhance personalized therapy selection [11]. Advanced AI frameworks, such as deep learning-based convolutional neural networks (CNNs) and transformer models, facilitate the identification of novel pharmacogenomic associations by analyzing high-dimensional data from multi-omics sources [12]. Additionally, AI-driven multi-omics integration strategies, such as the use of variational autoencoders (VAEs) and graph neural networks (GNNs), enable a more comprehensive understanding of drug response mechanisms in cancer [13]. However, the predictive accuracy of AI-based models is subject to constraints, including biases in training datasets, model interpretability, and generalizability across diverse patient populations. To ensure the clinical reliability of AI-driven pharmacogenomics, robust validation studies and standardized regulatory guidelines are required [14].

Several computational pipelines have been developed to streamline AI-driven pharmacogenomic analysis. For instance, the DeepCell framework employs deep learning for single-cell transcriptomic profiling to enhance cancer biomarker discovery [15]. Similarly, GNN leverages graph-based learning to predict drug response by incorporating gene expression and pathway-level interactions [16]. These AI-based approaches enhance the precision of drug selection by incorporating multi-layered biological insights, ultimately improving therapeutic decision-making.

Therapeutic strategies in precision oncology include targeted therapies, immunotherapies, and radiogenomics, which ensure tumor-specific interventions [17,18,19]. With continuous advancements, precision oncology is becoming integral to cancer care, improving survival rates and patient quality of life worldwide [20]. However, AI-driven pharmacogenomics still faces challenges related to algorithmic biases, explainability, and regulatory approvals, underscoring the necessity for transparent AI validation protocols and ethical frameworks to safeguard patient autonomy and data security [21,22].

### 1.3. The Role of Pharmacogenomics in Personalized Cancer Therapy

**Figure 1:**
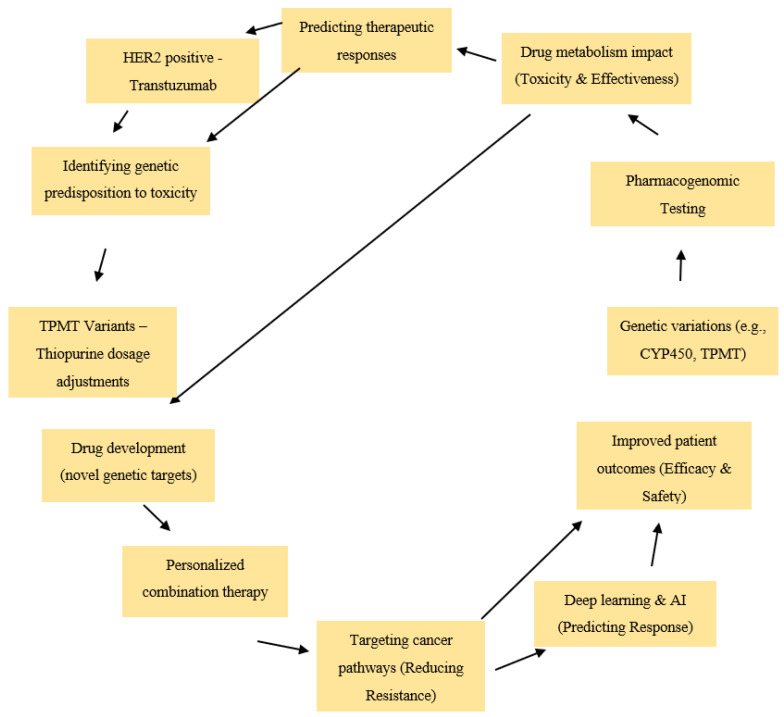
Role of pharmacogenomics in cancer therapy.

Pharmacogenomics (PGx) examines how genetic variations influence drug response, enabling personalized cancer therapies that optimize efficacy while minimizing toxicity (Figure 1) [23]. Variations in drug-metabolizing enzymes, particularly within the CYP450 family, significantly impact chemotherapy drug metabolism, altering both toxicity and effectiveness [24,25]. Genetic testing plays a critical role in predicting therapeutic response; for example, HER2-positive breast cancer patients benefit from trastuzumab (Herceptin) therapy due to their specific genetic profile [26]. Additionally, pharmacogenomics helps identify genetic predispositions to drug toxicity, such as thiopurine methyltransferase (TPMT) gene variants, which increase the risk of thiopurine-induced toxicity and necessitate dosage adjustments [27].

Beyond individual treatments, pharmacogenomics contributes to drug development by identifying novel genetic targets, leading to the creation of less toxic and more effective therapies. Personalized combination therapies, designed based on a tumor’s genetic and epigenetic profile, enable precise targeting of cancer pathways, enhancing treatment efficacy while reducing resistance risks [28,29]. Furthermore, deep-learning-based predictive modeling is now being applied to identify potential responders and non-responders to immunotherapy, thereby improving treatment stratification and clinical outcome [30]. The integration of pharmacogenomics into oncology is transforming treatment personalization, enhancing efficacy, minimizing toxicity, and ultimately improving patient outcomes. To ensure a comprehensive and systematic review, we conducted a literature search across PubMed, Scopus, Web of Science, and Google Scholar, using key terms such as “Circular RNA”, “circRNA”, “Pharmacogenomics”, and “Cancer Therapy” with Boolean operators. We included peer-reviewed articles published between 2015 and 2024, focusing on circRNA roles in drug metabolism, resistance, and precision medicine. Non-English studies, preprints, and unrelated papers were excluded. Relevant studies were selected based on title and abstract screening, followed by full-text evaluation. To facilitate comparative synthesis, the included studies were grouped according to cancer type, specific circRNAs investigated, and their pharmacogenomic roles such as diagnostic biomarkers, therapeutic targets, or modulators of drug resistance. This thematic classification allowed for a clearer analysis of circRNA functions across diverse cancer subtypes and mechanisms. No changes were made to the grouping strategy after the review was initiated, and no preregistered protocol was employed. This systematic approach helped synthesize emerging trends, mechanistic insights, and translational applications of circRNAs in oncology, ensuring transparency and reproducibility.

### 1.4. Circular RNAs: Emerging Players in Cancer Biology

circRNAs are a unique subclass of non-coding RNAs characterized by their covalently closed circular structure, which lacks 5′ caps and 3′ poly(A) tails. This configuration provides exceptional stability, rendering them resistant to ribonuclease degradation and extending their half-life compared to linear mRNAs [31]. Recent advances in circRNA-based liquid biopsies have demonstrated their potential as non-invasive biomarkers for detecting early-stage cancers and predicting therapy resistance, particularly in hematological malignancies and solid tumors [32]. circRNAs were first identified in 1976 as viroids in plants and later detected in human HeLa cells in 1979 [33,34]. Advances in RNA sequencing (RNA-seq) and bioinformatics have revealed their abundance in eukaryotic cells and their pivotal roles in diseases such as cancer, neurological disorders, and cardiovascular diseases [35,36].

circRNAs regulate gene expression at multiple levels, acting as modulators of transcription and translation, RNA-binding protein sponges, and microRNA (miRNA) sponges. As miRNA sponges, circRNAs function as competitive endogenous RNAs (ceRNAs), inhibiting miRNA activity. For instance, CDR1as (ciRS-7) contains over 70 conserved miR-7 binding sites, serving as an efficient sponge for miR-7 [36,37]. Despite these advancements, significant challenges remain in standardizing circRNA detection methods, as variations in expression across individuals and cancer subtypes raise concerns about their reproducibility in clinical applications [38]. Emerging research highlights circRNAs as potential RNA-based drug carriers, owing to their exceptional stability and tunable molecular properties [32].

### 1.5. Bridging Pharmacogenomics and circRNAs

CircRNAs play a crucial role in pharmacogenomics by influencing drug response, resistance, and therapeutic outcomes. Their unique properties, including high tissue specificity and differential expression in cancers, make them valuable biomarkers for assessing drug efficacy and toxicity. Certain circRNAs have been identified as promising diagnostic and prognostic biomarkers in non-small-cell lung cancer (NSCLC), highlighting their significance in understanding molecular characteristics across various cancer subtypes [39]. Recent studies have identified specific circRNAs as potential predictors of chemotherapy response in colorectal cancer. For instance, circHIF1A has been associated with cetuximab resistance, where its inhibition improves drug sensitivity and slows tumor growth [40]. Additionally, other circRNAs such as circCCDC66 (778 bp) have been implicated in modulating drug resistance mechanisms in colorectal cancer, highlighting their potential as biomarkers for personalized medicine approaches [41]. circRNAs also regulate genes involved in drug metabolism, transport, and cellular response pathways, directly impacting drug resistance. By modulating key biological processes such as cell growth and apoptosis, circRNAs influence the effectiveness of chemotherapy and targeted therapies [42].

circRNAs contribute to immune checkpoint modulation, which is essential for immunotherapy effectiveness. They regulate the activity of natural killer (NK) cells, T cells, and macrophages, making them potential targets or enhancers for immunotherapy. circCTNNB1 (370 bp) has been shown to modulate PD-L1 expression, promoting immune evasion in hepatocellular carcinoma [43]. Likewise, circARSP91 (91 bp) enhances NK cell-mediated immunity in liver cancer, offering potential as an immunotherapy adjuvant [44]. Their exceptional stability and tissue-specific expression further enhance their role as biomarkers for predicting treatment outcomes [45].

The pharmacogenomic potential of circRNA–miRNA–mRNA interactions is another key area of study. circRNAs act as competing endogenous RNAs (ceRNAs), forming complex regulatory networks with miRNAs and mRNAs. These networks help elucidate mechanisms underlying drug sensitivity and resistance. For instance, circHIPK3 (1099 bp) modulates the PI3K/AKT pathway, enhancing resistance to gefitinib in NSCLC, thus presenting a target for combination therapy [46]. Moreover, circRNAs influence the epigenetic regulation of key genes associated with cancer progression and drug response. Recent research indicates that circRNAs interact with chromatin remodelers like EZH2, influencing histone modifications that regulate drug resistance pathways [47]. In conclusion, integrating circRNA research with pharmacogenomics offers a novel framework for understanding individual variations in drug response and developing personalized therapeutic strategies. Despite their promise, challenges remain regarding reproducibility in biomarker validation, as sequencing variability affects circRNA standardization.

## 2. Pharmacogenomic Potential of Circular RNAs

### 2.1. circRNAs as Biomarkers for Drug Response

circRNAs have gained recognition as key regulators in cancer biology, particularly in influencing sensitivity and resistance to various therapeutic interventions. High-throughput sequencing and microarray analyses have identified circRNAs exhibiting differential expression in drug-resistant versus drug-sensitive cancer cell lines. In breast cancer, a microarray analysis identified 18 circRNAs differentially expressed between doxorubicin-resistant MCF-7/ADM cells and their doxorubicin-sensitive counterparts, with 12 upregulated and 6 downregulated in the resistant cells [48,49]. Recent studies have expanded this analysis, identifying circ_0006528 (496 bp) as a key player in doxorubicin resistance through miR-125b sponging, further regulating drug transporters such as ABCG2 [50]. Silencing circ_0006528 (496 bp) restored sensitivity to doxorubicin, suggesting its role in mediating drug resistance [51]. Similar findings have been reported for docetaxel and cisplatin resistance, with circRNAs such as circATP8B4 (669 bp) and circCD44 (357 bp) being linked to variations in drug sensitivity, further reinforcing the role of circRNAs in chemotherapy response [52,53].

circRNAs modulate drug resistance through multiple mechanisms, including miRNA sponging, protein interactions, and gene regulation. As miRNA sponges, circRNAs bind and sequester miRNAs, preventing them from suppressing target mRNAs and thereby promoting drug resistance pathways [54,55]. For instance, circCYP24A1 (1106 bp) has been implicated in cisplatin resistance in lung cancer through miR-130b-5p sponging and modulation of the Wnt/β-catenin pathway [56]. Additionally, circRNAs interact with proteins, modulating their activity and stability to influence cell survival and drug response. Certain circRNAs regulate apoptosis and cell cycle checkpoints, leading to chemoresistance by reducing drug-induced apoptosis [57,58]. Moreover, circRNAs influence gene transcription by interacting with RNA polymerase II or transcription factors, thereby altering the expression of genes involved in drug metabolism and resistance.

Due to their specificity, stability, and resistance to exonucleases, circRNAs are emerging as promising biomarkers for predicting therapeutic response. However, the lack of standardization in circRNA detection and quantification remains a challenge. Recent advances in machine learning and bioinformatics pipelines have enabled the development of circRNA-based predictive models for chemotherapy response, aiding in patient stratification for personalized treatments [59]. Future efforts should focus on validating circRNA-based biomarkers across diverse patient populations, incorporating multi-omics approaches to enhance reliability and standardization in clinical applications.

### 2.2. circRNA Expression Variability and Pharmacogenomic Profiling

Inter-individual variability in circRNA expression across different populations and tissues is influenced by genetic, environmental, and technical factors. Genetic differences play a significant role in circRNA expression regulation. Studies have identified specific quantitative trait loci (QTLs) associated with circRNA levels, termed circQTLs, which indicate that individual genetic variations contribute to circRNA expression variability [60]. Furthermore, genome-wide association studies (GWAS) have revealed population-specific circQTLs that affect cancer susceptibility and treatment response [61].

Tissue specificity is another major determinant of circRNA expression. A comprehensive analysis of human liver samples identified 59,128 potential circRNA candidates, with nearly 89% present in only one or two samples, demonstrating significant inter-individual variation. In contrast, approximately 10% of linear mRNAs and non-coding RNAs were detected consistently across all samples, reflecting more uniform expression. Similarly, an analysis of brain tissues identified 50,631 circRNAs, with only 0.5% consistently expressed across 30 brain samples, highlighting substantial tissue-specific variability [62,63].

Despite their promise as pharmacogenomic biomarkers, circRNA-based approaches face significant limitations in reproducibility, standardization, and cross-study validation. Differences in RNA extraction techniques, library preparation, and sequencing depth lead to technical variability, affecting the identification and quantification of circRNAs [64]. Additionally, inconsistencies in bioinformatics pipelines, including circRNA annotation tools and normalization strategies, further hinder reproducibility across studies [65]. Several reports highlight discrepancies in circRNA detection due to variations in sequencing platforms, with some circRNAs identified in one study but absent in others analyzing the same tissue type [66].

Furthermore, environmental and biological factors, such as age, sex, and disease state, influence circRNA expression, complicating their role as universal biomarkers [58]. For instance, age-related declines in circRNA expression have been linked to alterations in drug metabolism pathways, affecting their reliability in elderly populations [67]. Machine-learning-assisted normalization of RNA-seq data has been proposed as a solution to improve data comparability across studies, but further efforts are needed to establish standardized methodologies and validate circRNA-based biomarkers through large-scale, multi-center clinical trials [68,69].

### 2.3. circRNAs in Modulating Drug Metabolism and Transport

circRNAs play a pivotal role in drug metabolism by regulating cytochrome P450 (CYP) enzymes and ABC transporters, both of which are critical determinants of drug response and resistance. The CYP enzyme family, which includes CYP3A4, CYP2C19, and CYP2D6, is responsible for the metabolism of more than 75% of clinically prescribed drugs. Recent findings have identified circRNAs derived from CYP genes that influence drug metabolism, particularly in hepatocytes, where circCYP2C19 modulates warfarin metabolism [70,71].

circRNAs also regulate ATP-binding cassette (ABC) transporters, which play a crucial role in multidrug resistance (MDR) by promoting drug efflux. CircABCB1 (543 bp) was shown to enhance P-glycoprotein-mediated drug efflux, contributing to doxorubicin resistance in leukemia [72]. circRNAs function as competing endogenous RNAs (ceRNAs), influencing the expression of ABC transporters by binding to miRNAs that typically suppress ABC transporter mRNA, preventing miRNA-mediated repression and thereby enhancing transporter expression. This mechanism contributes to chemotherapy failure by reducing intracellular drug accumulation.

Although circRNAs hold potential as pharmacogenomic biomarkers, there remain critical gaps in their clinical application. Efforts to standardize circRNA-based assays, develop cost-effective detection technologies, and validate findings in large-scale clinical trials are needed to facilitate their translation into clinical practice. Additionally, AI-driven multi-omics integration could enhance the identification of circRNA signatures associated with drug metabolism and resistance, providing new avenues for personalized cancer therapies [73].

## 3. CircRNA-Mediated Drug Resistance in Cancer

### 3.1. Mechanistic Insights into circRNA-Driven Drug Resistance

**Figure 2:**
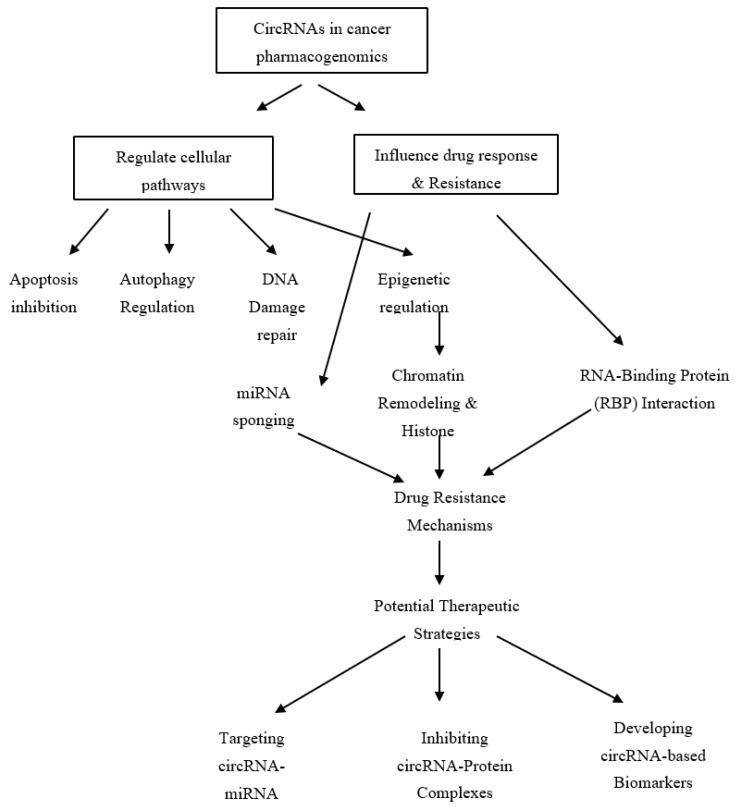
CircRNA-mediated drug resistance in cancer.

circRNAs regulate multiple cellular pathways, including apoptosis, autophagy, and DNA repair, which contribute to tumor progression and therapy resistance (Figure 2). These regulatory functions make circRNAs promising targets for overcoming drug resistance in cancer therapy. One of the key ways in which circRNAs promote therapy resistance is by modulating cell death pathways. For example, circ_0001955 (815 bp) inhibits ferroptosis, a non-apoptotic form of cell death, by reducing lipid peroxidation and enhancing resistance to chemotherapy in hepatocellular carcinoma [74,75]. Similarly, circ-MAPK4 (259 bp), detected in clinical glioma samples, promotes tumor progression by preventing apoptosis through the suppression of caspase 3/7/9 activation and enhancing p38/MAPK signaling, thereby increasing glioma resistance to therapy [76].

Autophagy is another key mechanism by which circRNAs contribute to drug resistance, with specific circRNAs regulating autophagy genes via miRNA sponging and protein interactions. circCDYL, for instance, modulates autophagy by sponging miR-1275, which regulates ULK1 and ATG7, two key players in the initiation of autophagy. This interaction promotes breast cancer chemoresistance, making autophagy a potential therapeutic target for overcoming resistance [77,78]. circRNAs also influence DNA damage repair (DDR), which plays a crucial role in cancer cell survival under genotoxic stress. For instance, circRAD18 enhances homologous recombination repair, increasing resistance to cisplatin in bladder cancer [79,80]. Similarly, circAKT3 (524 bp) promotes DNA damage repair in gastric cancer by sponging miR-198, which suppresses PIK3R1 expression, thereby activating the PI3K/AKT pathway [81].

Given the overlapping roles of apoptosis, autophagy, and DNA repair in circRNA-mediated resistance, a comparative analysis of these pathways would improve our understanding of their relative contributions. Understanding how these mechanisms interact could provide insights into novel combination therapies that target multiple resistance pathways simultaneously.

### 3.2. circRNAs as miRNA Sponges in Drug Resistance

One of the most well-documented mechanisms by which circRNAs mediate drug resistance is their role as miRNA sponges. circRNAs contain multiple miRNA response elements (MREs) that allow them to sequester miRNAs, preventing them from suppressing oncogenic targets. By acting as competing endogenous RNAs (ceRNAs), circRNAs disrupt miRNA-mediated gene silencing and promote the expression of drug resistance-related genes.

For instance, circPVT1 sponges miR-423-5p, enhancing paclitaxel resistance in ovarian cancer [78]. Similarly, circFOXO3 (1435 bp) sponges miR-23a-3p, preventing it from down regulating PTEN, which in turn enhances cisplatin resistance in lung cancer [79]. circHIPK3 (1099 bp) interacts with miR-124, modulating ABC transporter proteins, increasing drug efflux, and diminishing chemotherapy efficacy in breast cancer [80]. The functional impact of these circRNAs is influenced by their size, as circRNA prediction may identify multiple regions from the same gene, with variations in size affecting their ability to function as miRNA sponges. Additionally, the sequence of each circRNA determines the specific regions that can interact with miRNAs, influencing their role in drug resistance [81,82].

While miRNA sponging is well-described, circRNAs also interact with RNA-binding proteins (RBPs), which play a crucial role in modulating gene expression and protein stability. For example, circ_0003418 (504 bp) interacts with chromatin remodelers, thereby altering epigenetic modifications that regulate drug resistance genes [83] (Figure 2). These circRNA-protein interactions can affect post-translational modifications, chromatin remodeling, and gene accessibility, contributing to drug resistance. Expanding the discussion to include these interactions provides a more holistic view of how circRNAs mediate therapy resistance.

### 3.3. circRNAs and Epigenetic Regulation in Drug Resistance

Emerging evidence suggests that circRNAs influence gene regulation via epigenetic mechanisms, including chromatin remodeling and histone modifications. These processes allow tumors to adapt dynamically to drug treatments, leading to resistance. By altering chromatin accessibility, histone acetylation, and DNA methylation, circRNAs contribute to a more plastic cancer genome, enabling cells to survive and evade therapy.

circRNAs directly interact with chromatin-modifying proteins, thereby altering histone methylation and acetylation. For instance, circSMARCA5 (269 bp) recruits the histone acetyltransferase KAT7, enhancing H3 acetylation and promoting oncogene expression in glioblastoma [84]. Similarly, circRNA_002178 (617 bp) regulates the EZH2/PRC2 complex, contributing to gastric cancer resistance through histone H3K27me3 modifications [85]. These findings suggest that circRNAs not only regulate transcription post-transcriptionally but also affect chromatin modifications, altering the epigenetic landscape of drug-resistant tumors.

Despite their potential as biomarkers for therapy resistance, standardization challenges remain a major barrier to clinical implementation. Inter-individual variability in circRNA expression is influenced by factors such as ethnicity, age, and environmental exposures, complicating their reliability as universal biomarkers [86] (Figure 2). Moreover, discrepancies in sequencing technologies, bioinformatics pipelines, and normalization strategies hinder reproducibility across studies. Variations in RNA isolation methods, sequencing depth, and circRNA annotation tools further contribute to inconsistencies in expression profiling, limiting the translational potential of circRNAs in precision oncology [66].

To overcome these limitations, standardized sequencing protocols and robust bioinformatics pipelines are needed to ensure accurate circRNA detection and quantification [87]. Machine learning models and AI-driven computational approaches have shown promise in normalizing circRNA expression data across different patient populations, enhancing reproducibility and predictive accuracy [88]. Additionally, large-scale multi-center clinical trials are essential to validate circRNA-based biomarkers and establish their clinical significance in drug resistance and personalized therapy [89].

## 4. circRNAs as Therapeutic Targets in Cancer

### 4.1. CircRNA-Based Therapies for Overcoming Drug Resistance

Targeting circRNAs presents a promising strategy for counteracting drug resistance in cancer therapy. Two primary approaches-antisense oligonucleotides (ASOs) and CRISPR/Cas systems—have been explored to regulate circRNA expression. ASOs are synthetic short nucleic acid sequences that hybridize with target RNA molecules, leading to their degradation or functional inhibition. In the context of circRNAs, ASOs can be used to reduce their expression levels, thereby mitigating their role in drug resistance [90]. Studies have demonstrated that ASO-mediated knockdown of circHIPK3 (1099 bp) restores chemosensitivity in colorectal cancer by modulating the miR-124/IGF2BP2 axis, leading to reduced oncogenic signaling [91].

A specialized ASOs known as a gapmer, which consists of a central DNA region flanked by modified RNA nucleotides, has been particularly effective in targeting upregulated circRNAs. The central DNA region induces RNase H-mediated degradation of the target RNA, while the modified flanking regions enhance binding affinity and protect against exonuclease degradation [92]. A gapmer-based silencing of circCDR1 (1500 bp) as in hepatocellular carcinoma has improved sorafenib sensitivity by modulating miR-7/PTEN signaling [93]. Similarly, ASO-mediated circRNA knockdown in breast cancer has decreased chemoresistance, highlighting the potential of this approach in enhancing therapeutic efficacy [94].

CRISPR/Cas technology has introduced more flexible and precise RNA-targeting strategies. The CRISPR/Cas9 system is widely known for genome editing, but its ability to disrupt circRNA biogenesis by deleting or modifying back-splice junctions or intronic sequences has expanded its therapeutic applications [95,96]. In contrast, CRISPR/Cas13 selectively targets and cleaves circRNAs at the RNA level, making it an effective tool for post-transcriptional regulation. Studies have shown that CRISPR/Cas13-mediated depletion of circABCB1 (543 bp) suppresses multidrug resistance in leukemia cells by downregulating ABC transporter expression. Additionally, CRISPR/Cas13-mediated silencing of circPVT1 in ovarian cancer enhanced paclitaxel sensitivity by disrupting miR-423-5p sponging and restoring FOXO3 expression [97]. These findings demonstrate that CRISPR-based strategies can selectively modulate circRNA levels without affecting host gene expression, making them highly promising tools for cancer therapy [96,98].

Effective delivery of antisense oligonucleotides (ASOs) targeting circRNAs is crucial for overcoming drug resistance in cancer therapy. Lipid-based nanoparticles (LNPs) have emerged as promising carriers for ASOs, enhancing their stability and cellular uptake. For instance, studies have shown that LNP-encapsulated ASOs exhibit increased activity compared to non-encapsulated counterparts, although challenges remain in achieving widespread distribution in vivo [99]. Additionally, exosomes natural extracellular vesicles have been engineered to deliver ASOs effectively to tumor sites, offering targeted delivery with reduced immunogenicity. These advancements in delivery systems are pivotal for translating circRNA-targeting strategies into clinical applications. The use of polymeric and peptide-based nanoparticles has further enhanced delivery efficiency, enabling the controlled release of ASOs and siRNAs targeting circRNAs [100]. These advancements in delivery systems are pivotal for translating circRNA-targeting strategies into clinical applications.

### 4.2. Synthetic circRNAs for Drug Delivery

Synthetic circRNAs have gained interest as potential tools for therapeutic applications due to their unique structure, stability, and resistance to exonucleases. Their closed-loop configuration provides an extended half-life, making them suitable for delivering therapeutic agents or modulating biological pathways. Recent studies have demonstrated that synthetic circRNAs engineered to sponge oncogenic miRNAs suppress tumor growth and improve drug sensitivity in preclinical models of lung and breast cancer [101,102].

Synthetic circRNAs can be produced using three main methods: chemical synthesis, enzymatic ligation, and ribozyme-based circularization. Chemical synthesis allows for precise sequence control and modifications, but it is limited by length constraints. Enzymatic ligation and ribozyme-based circularization have emerged as scalable techniques, producing long and functionally optimized synthetic circRNAs [103,104]. Each method has distinct advantages in efficiency, stability, and cost, necessitating further optimization for clinical translation.

Beyond functioning as delivery vehicles, synthetic circRNAs modulate specific cellular pathways by acting as molecular sponges for miRNAs or regulatory scaffolds for protein interactions. For instance, synthetic circRNAs designed to inhibit miR-21, a key oncogenic miRNA associated with chemoresistance, enhance apoptosis and improve chemotherapy efficacy in colorectal cancer [102]. Additionally, circRNAs have been engineered to facilitate protein complex formation, influencing proliferation, apoptosis, and immune responses [105].

The unique size and structure of circRNAs make them suitable candidates for incorporation into various delivery mechanisms, including nanocarriers, liposomes, and extracellular vesicles. Preclinical studies have demonstrated that synthetic circRNAs can be effectively delivered using these platforms. For example, lipid-based nanoparticles have been utilized to deliver synthetic circRNAs targeting oncogenic pathways, resulting in suppressed tumor growth in animal models [106]. Similarly, exosome-mediated delivery of synthetic circRNAs has shown promise in achieving targeted therapy with minimal off-target effects [107]. These findings underscore the potential of leveraging nanocarriers and extracellular vesicles for the efficient and targeted delivery of therapeutic circRNAs.

### 4.3. CircRNA Modulation in Combination Therapies

Understanding the synergistic effects of circRNA modulation in combination with chemotherapy, targeted therapy, and immunotherapy has opened new avenues for improving cancer treatment efficacy. circRNAs influence cancer cell sensitivity to chemotherapy drugs, and targeting them could enhance existing treatment regimens. For example, circ_0001658 (48071 bp) contributes to resistance against EGFR inhibitors by modulating TWIST1 expression, suggesting a potential therapeutic target for improving NSCLC response to targeted therapy [40].

circRNAs also mediate resistance to targeted therapies by interacting with RNA-binding proteins and miRNAs. circHIPK3 (1099 bp) enhances PI3K/Akt signaling, contributing to resistance to PI3K inhibitors in breast cancer, making it a promising target for combination therapy [108]. These interactions underscore the role of circRNAs in modulating key oncogenic pathways, reinforcing the need to develop therapeutic strategies that inhibit circRNA-mediated resistance mechanisms.

Emerging research indicates that circRNAs influence immune evasion mechanisms, making them potential targets for immunotherapy sensitization [109]. A pioneering study demonstrated that combining Piwi-interacting RNAs (piRNAs) and circRNAs enhances the efficacy of PD-1/PD-L1 inhibition therapy, providing a novel strategy to improve immune checkpoint blockade responses [110]. Additionally, circPD-L1 has been shown to regulate PD-L1 expression, impacting the efficacy of immunotherapy in melanoma. Recent preclinical trials suggest that targeting circRNAs involved in immune evasion, such as circARSP91 (91 bp), can enhance NK cell-mediated tumor immunity, presenting a novel avenue for improving immunotherapeutic outcomes [111].

Thus, circRNA modulation represents a promising strategy to enhance the effectiveness of chemotherapy, targeted therapy, and immunotherapy. By identifying circRNA simplicated in therapy resistance, researchers may develop synergistic treatment regimens that improve patient outcomes and pave the way for precision oncology approaches.

## 5. Emerging Tools and Techniques for circRNA-Based Pharmacogenomics

### 5.1. High-Throughput Technologies for circRNA Profiling

The evolution of high-throughput sequencing technologies has significantly improved the detection and characterization of circular RNAs (circRNAs), particularly in pharmacogenomics. Initially, traditional methods such as Northern blotting and RT-PCR were used for circRNA detection; however, these approaches had limitations in specificity, scalability, and the ability to distinguish circRNAs from linear RNAs [112]. The advent of high-throughput RNA sequencing (RNA-seq) revolutionized circRNA detection by enabling unbiased, transcriptome-wide identification. This advancement led to the development of computational tools such as CIRI, Find_circ, and circRNA_finder, which enhanced circRNA annotation and prediction [36,113]. Notably, studies by Memczak et al. (2013) and Jeck et al. (2013) were among the first to demonstrate the widespread existence of circRNAs using RNA-seq, providing key insights into their stability and functional relevance [33,114].

More recently, single-molecule sequencing technologies, including Nanopore, have emerged, offering long-read capabilities that allow for the direct detection of full-length circRNA isoforms without the need for PCR amplification. This reduces amplification bias and improves structural annotation accuracy [115,116,117]. Compared to short-read sequencing, nanopore sequencing provides higher accuracy in identifying circRNA isoforms and alternative splicing events, which is crucial for understanding circRNA functional diversity [37]. Similarly, scRNA-seq enables the identification of cell-type-specific circRNA expression, providing insights into circRNA heterogeneity across tissues and disease states [113].

In addition, AI-enhanced circRNA-specific microarrays have been developed to improve the sensitivity of low-abundance circRNA detection, making them valuable for large-scale biomarker discovery [118]. While RNA-seq provides comprehensive transcriptomic data, circRNA microarrays offer a cost-effective alternative for large-scale biomarker screening in clinical settings. However, a major challenge in circRNA research is the variability in annotation pipelines. Differences in sequencing depth, reference genome versions, and computational algorithms often lead to discrepancies in circRNA datasets, making cross-study reproducibility difficult [119].

Although PCR-based validation techniques, such as quantitative real-time PCR (qRT-PCR) and Northern blotting, remain essential for confirming circRNA expression, they are not sufficient to fully characterize circRNA isoforms or reliably detect back-splice junctions [37,120]. Additional validation methods, including RNase R treatment (which selectively degrades linear RNA), Sanger sequencing (to confirm back-splice junctions), and functional studies such as circRNA knockdown and overexpression, provide more robust confirmation of circRNA identity and function [121,122,123]. The integration of machine learning models has further enhanced circRNA annotation, but their accuracy depends on the availability of large-scale, standardized datasets [124]. Establishing unified circRNA detection pipelines and robust validation strategies will be crucial for ensuring reproducibility and improving the reliability of circRNA-based studies.

### 5.2. circRNA-Pharmacogenomic Databases and Resources

The growing recognition of circRNAs in pharmacogenomics has led to the development of specialized databases that catalog circRNA-drug interactions and provide functional annotations. CircPharmaDB is one such resource that integrates circRNA-drug response data, allowing researchers to investigate how circRNAs influence drug resistance mechanisms and treatment responses in precision medicine [125]. While CircBase remains the most widely used circRNA repository, it lacks pharmacogenomic-specific annotations, limiting its utility for drug interaction studies [126]. Other resources such as NcRNADrug focus on validated and predicted circRNA-drug interactions, particularly in the context of drug resistance and targeted therapies [127]. AI-powered platforms like CircTheraScan leverage deep learning models to predict circRNA-drug interactions in real time, accelerating the discovery of novel pharmacogenomic targets [128].

#### 5.2.1. Comparative Analysis of circRNA Databases

A comparison of the databases based on their reliability, validation methods, and focus on drug response is summarized in Table 1. Below, we highlight the strengths and limitations of each database and their relevance to pharmacogenomics research:

#### 5.2.2. Reliability and Validation Considerations

Despite these advances, standardization remains a major challenge. CircRNA datasets vary across different platforms, leading to inconsistencies in pharmacogenomic annotations. While some databases, such as NcRNADrug and CircRiC, rely on experimental validation, others incorporate computationally predicted interactions, which can introduce biases [128,133]. The integration of machine learning-based predictive models, such as those used in CircTheraScan and GATECDA, holds promise but requires extensive validation with experimental data [113,120].

Harmonization efforts between different circRNA-pharmacogenomic databases could improve data consistency and enhance reproducibility, particularly in predicting drug responses based on circRNA profiles [128]. Establishing benchmark datasets and standardized validation protocols will be crucial for improving the clinical utility of circRNA-targeted therapies [135]. As circRNA research continues to expand, efforts to integrate experimental data with predictive models will play a key role in advancing precision medicine applications. Large-scale, multi-center studies are essential to validate circRNA-based pharmacogenomic biomarkers and establish their clinical relevance.

### 5.3. Experimental Models for circRNA Functional Validation

Experimental validation is crucial for confirming the functional role of circRNAs in drug resistance and pharmacogenomics. Several model systems, including CRISPR-based gene-editing tools, patient-derived xenografts (PDXs), and organoid models, have been developed to assess circRNA functions in drug response. CRISPR-Cas13 systems have emerged as a powerful tool for RNA targeting, enabling researchers to selectively degrade circRNAs and evaluate their impact on drug sensitivity [136]. Compared to ASO-mediated knockdown, which relies on RNase H degradation, CRISPR-Cas13 provides a more precise and programmable approach for circRNA depletion [137]. PDX models have been widely used to study drug resistance mechanisms in vivo, as they maintain the genetic and histological characteristics of patient tumors, making them an ideal model for assessing the effects of circRNA-targeted interventions on drug response [138,139]. However, PDXs exhibit significant inter-patient variability, which limits reproducibility across studies.

Organoid models offer a scalable and rapid in vitro alternative for studying circRNA functions, allowing researchers to test multiple drug combinations in a controlled environment. While organoids lack the complexity of the tumor microenvironment found in PDX models, they enable high-throughput drug screening, making them valuable for pharmacogenomic research [140]. The choice of experimental model depends on the research question-while PDXs provide in vivo relevance, organoids are more suitable for large-scale drug testing. Despite these advances, standardization challenges remain in functional validation studies. The variability in circRNA expression across patient-derived models underscores the need for standardized protocols to improve reproducibility [141,142]. Developing unified experimental guidelines for circRNA knockdown, validation, and pharmacogenomic analysis will be critical for translating circRNA research into clinical applications.

## 6. Clinical Translation of circRNAs in Pharmacogenomics

### 6.1. circRNAs as Predictive Biomarkers in Cancer Therapy

circRNAs have demonstrated immense potential as biomarkers for therapy selection due to their tissue-specific expression and involvement in disease-related pathways. Recent studies indicate that circRNA signatures can predict patient response to immune checkpoint inhibitors (ICIs), thus improving patient stratification in immunotherapy (Table 2) [68]. In colorectal cancer, certain circRNAs have been identified as diagnostic and prognostic biomarkers, influencing treatment strategies and improving precision oncology approaches.

The expression levels of specific circRNAs correlate with treatment responses, making them valuable for predicting clinical outcomes. For instance, circHIPK3 (1099 bp) has been identified as a non-invasive biomarker for chemotherapy resistance in bladder cancer, correlating with cisplatin resistance [143]. Here are some studies highlighting the role of circRNAs as predictive biomarkers for drug response in various cancers.

**Table 2:** circRNAs as predictive biomarkers for drug response in cancer therapy.

circRNA (ID, Size)	Cancer Type	Proposed Clinical Role	Reference *
circHIPK3 (hsa_circ_0000284; 1099 bp)	Gastric Cancer	Diagnostic biomarker for cisplatin resistance; therapeutic target to enhance ferroptosis	[144]
circATIC (hsa_circ_0058063; 1640 bp)	Bladder Cancer	Diagnostic biomarker for cisplatin resistance; therapeutic target to modulate miR-335-5p/B2M axis	[145]
circMORC3 (hsa_circ_0001189; 420 bp)	Bladder Cancer	Therapeutic target to affect m6A modification on DNA damage response genes	[146]
circPVT1 (hsa_circ_0001821; 410 bp)	Osteosarcoma	Diagnostic biomarker and therapeutic target for doxorubicin and cisplatin resistance	[147]
circ-ABCB10 (hsa_circ_0008717; 724 bp)	Lung Cancer	Therapeutic target to enhance cisplatin sensitivity	[148]
circSMARCA5 (hsa_circ_0001445; 269 bp)	Prostate Cancer	Inhibits tumor proliferation, migration, and invasion by regulating the miR-181b-5p/miR-17-3p-TIMP3 axis	[149]
circELP3 (hsa_circ_0001785; 467 bp)	Breast Cancer	Potential therapeutic target; involved in tumor progression through miRNA sponging	[150]
circCNIH4 (hsa_circ_0000190; 254 bp)	Colorectal Cancer	Diagnostic biomarker; upregulated in tissues and plasma	[151]

* References [144,145,146,147,148,149,150,151] correspond to citation numbers in the main manuscript.

Despite their promise, several challenges hinder the clinical implementation of circRNAs as predictive biomarkers. Variability in circRNA expression among individuals, tumor subtypes, and sequencing methodologies limits clinical reproducibility. Additionally, standardizing circRNA-based diagnostic platforms is critical to ensure consistent and reliable biomarker applications. Advances in liquid biopsy technology, particularly lipid nanoparticle (LNP) and exosome-based circRNA delivery systems, are addressing some of these limitations, providing new avenues for non-invasive cancer monitoring [152].

### 6.2. circRNAs in Personalized Therapeutic Regimens

The integration of circRNA profiling into personalized medicine presents significant opportunities for improving cancer diagnosis and treatment optimization. circRNAs offer high stability, abundance, and distinct expression patterns, making them ideal candidates for precision medicine. Recent advancements in multi-omics integration, which combines circRNA data with proteomics and metabolomics, have improved predictive accuracy for treatment responses, facilitating biomarker discovery [153].

Beyond diagnostics, circRNAs play a crucial role in therapeutic monitoring and treatment adjustments. Their expression levels reflect disease progression and treatment responses, allowing clinicians to modify therapeutic regimens based on circRNA-guided predictions. For instance, tracking circFOXO3 (1435 bp) levels has been linked to HER2-targeted therapy efficacy, enabling personalized dose optimization [154].

CircRNAs function as microRNA sponges, modulating gene expression linked to drug resistance and metabolism. These interactions influence the effectiveness of chemotherapy and targeted therapies, making them valuable pharmacogenomic markers for therapy customization. Additionally, the stability of circRNAs in bodily fluids supports their use in liquid biopsies, enabling non-invasive therapy monitoring and timely treatment adjustments.

### 6.3. Challenges and Opportunities for Clinical Applications

Despite their promise, several challenges must be addressed before circRNAs can be fully integrated into clinical practice. One of the major barriers is the lack of standardized circRNA reference databases, as discrepancies between different datasets hinder biomarker validation and clinical reproducibility [155].

Another critical issue is scalability and cost-effectiveness. Recent advances in enzymatic synthesis and chemical ligation have improved large-scale circRNA production efficiency, but challenges remain in achieving high-purity, functionally active circRNAs for therapeutic applications [156]. Ensuring proper circularization and biological function is crucial for clinical translation.

circRNAs exhibit tissue-specific expression and are detectable in blood, urine, and saliva, making them attractive biomarkers for early cancer detection and therapeutic monitoring. Recent studies highlight that circRNA-based liquid biopsies outperform traditional mRNA biomarkers, demonstrating higher sensitivity and specificity for early cancer detection [157].

However, regulatory concerns remain a major challenge. Given that circRNA-based therapies are still in early-stage clinical trials, regulatory bodies such as the FDA and EMA require extensive validation to ensure safety, stability, and minimal off-target effects. Addressing these regulatory and standardization issues is essential to successfully integrate circRNA-based interventions into clinical oncology.

## 7. Future Perspectives in circRNA Pharmacogenomics

### 7.1. Toward Personalized Medicine: The Role of circRNAs

circRNAs exhibit tissue-specific expression patterns and differential regulation across various cancer types, including lung, gastrointestinal, and urological cancers. These unique expression profiles position circRNAs as promising biomarkers for early cancer detection, diagnosis, and prognosis. Recent studies indicate that integrating circRNA profiling into machine-learning-driven diagnostic tools has significantly improved early cancer detection accuracy [158]. However, further validation studies are required to compare the performance of circRNA-based classifiers with conventional liquid biopsy biomarkers such as circulating tumor DNA (ctDNA) or protein markers.

Emerging research suggests that circRNAs play a crucial role in tumor immunity and may be leveraged in immunotherapeutic approaches. For instance, circPD-L1 has been found to modulate PD-L1 expression, affecting immune checkpoint blockade therapy outcomes in melanoma [159]. While this discovery presents an exciting avenue for precision oncology, comparative studies with existing PD-L1 inhibitors are needed to assess whether circRNA modulation enhances or merely complements current strategies. Furthermore, circRNA-based vaccines are being explored for personalized immunotherapy, particularly in targeting tumor-specific antigens. However, their efficacy, immunogenicity, and safety compared to mRNA-based vaccines remain unexplored.

circRNAs are also being explored as therapeutic targets due to their stable structure and selective expression in cancer cells, making them promising candidates for targeted therapeutic interventions. Recent advancements in circRNA-engineered CAR-T cell therapy suggest that circRNA modulation can improve anti-tumor specificity while reducing off-target effects [160]. However, more studies are needed to compare the functional advantages of circRNA-modified CAR-T cells against traditional CAR-T approaches, particularly regarding persistence, expansion potential, and tumor penetration. Overall, circRNAs hold substantial promise in refining personalized oncology, but rigorous clinical validation is crucial before widespread clinical adoption.

### 7.2. Unexplored Frontiers in circRNA Pharmacogenomics

Exploring the intersections of rare cancers, combinatorial drug effects, and population-specific circular RNAs presents valuable opportunities for advancing cancer research and treatment. Recent transcriptomic analyses of rare cancer subtypes have revealed unique circRNA signatures, providing novel insights into tumor biology and potential therapeutic targets [161]. However, comparative studies examining whether circRNAs perform better than traditional genetic biomarkers (e.g., TP53 or BRCA mutations) in rare cancers are still lacking.

circRNAs are increasingly recognized for their role in drug resistance and their potential as therapeutic targets in combination therapies. For example, circ_0003418 (504 bp) has been identified as a regulator of drug metabolism pathways in pancreatic cancer, making it a promising target for combination therapy strategies [162]. Further research into circRNA-drug interactions may inform the design of combinatorial treatments, potentially overcoming resistance and improving patient outcomes.

Population-specific studies are essential for uncovering the genetic and molecular factors influencing diseases across diverse groups. Variations in circRNA expression can arise due to genetic diversity, environmental influences, and lifestyle differences. Studies focusing on ethnically diverse patient cohorts have highlighted the role of circRNA polymorphisms in differential drug responses, paving the way for personalized therapeutics [163]. While circRNAs hold promise in pharmacogenomics, further studies are required to standardize experimental methods and validate findings across different cancer types and ethnic backgrounds. Future directions should explore integrative multi-omics approaches, leveraging AI-driven analyses and longitudinal cohort studies to establish circRNAs as clinical biomarkers and therapeutic targets.

### 7.3. Bridging Basic Research and Clinical Practice

Recent preclinical studies have explored various approaches to harness circRNAs for therapeutic applications. Advancements in RNA synthesis have enabled the development of synthetic circRNAs capable of modulating gene expression. Notably, synthetic circRNAs have been engineered to act as miRNA sponges in glioblastoma, successfully suppressing tumor progression in preclinical models [164]. However, concerns regarding stability, immune activation, and delivery efficiency must be addressed before these molecules can transition into human trials.

Due to their stability and ability to maintain antigen expression, circRNAs have been investigated as promising platforms for vaccine development. A circRNA-based vaccine targeting viral oncoproteins has shown robust immune activation and tumor suppression in HPV-associated cancers, underscoring the potential for broader oncologic applications [165]. Despite these promising findings, circRNA vaccines need to be evaluated alongside existing nucleic acid-based vaccines (e.g., mRNA, DNA-based platforms) to determine their relative efficacy and immune activation potential.

One of the most promising clinical applications of circRNAs is non-invasive liquid biopsy-based diagnostics. circRNA profiling in blood, saliva, and urine has demonstrated higher stability and specificity than protein-based biomarkers. Recent studies suggest that circRNA-based liquid biopsies outperform ctDNA panels in early-stage colorectal cancer detection, demonstrating their potential for early intervention strategies [166,167]. However, standardization and reproducibility remain key challenges, as circRNA detection methods lack uniformity across different platforms.

The regulatory pathway for circRNA-based therapeutics and diagnostics remains undefined, posing a significant hurdle for clinical translation. Unlike small-molecule drugs and monoclonal antibodies, RNA-based therapeutics require distinct stability, immune safety, and delivery efficiency considerations. Addressing these regulatory challenges will require collaborations between researchers, clinicians, and policymakers to establish robust clinical trial frameworks and ensure standardized circRNA biomarker validation methodologies.

## 8. Conclusions

CircRNAs are emerging as critical regulators in pharmacogenomics, profoundly influencing drug response, metabolism, transport, and therapeutic efficacy. Their stability, tissue-specific expression, and functional versatility position them as pivotal players in precision medicine. In addition to their regulatory functions, circRNAs demonstrate significant potential as predictive biomarkers, providing clinicians with enhanced tools for therapy selection and patient stratification. By modulating key signaling pathways, circRNAs influence drug resistance, impact targeted therapy outcomes, and shape immunotherapy responses, making them valuable assets in personalized oncology.

Advancements in sequencing technologies, bioinformatics, and gene-editing techniques have accelerated circRNA research, uncovering their diverse roles in tumor progression and therapy resistance. Their ability to function as both oncogenic drivers and tumor suppressors highlights their dual role in cancer biology. Emerging therapeutic strategies, including CRISPR-mediated circRNA modulation, synthetic circRNA delivery systems, and their integration into chimeric antigen receptor (CAR)-T cell therapy, offer new avenues for targeted cancer treatment. These developments underscore the potential of circRNA-based therapies in overcoming drug resistance and enhancing treatment efficacy.

Recent discoveries have expanded the therapeutic landscape of circRNAs, not only as molecular regulators but also as platforms for drug delivery and vaccine development. CircRNA-based liquid biopsies present new possibilities for non-invasive cancer diagnostics, enabling real-time monitoring of disease progression and therapeutic response. Furthermore, population-specific circRNA profiles and their role in pharmacogenomics highlight the necessity of personalized treatment approaches that account for genetic and environmental variations across diverse patient populations.

Despite these advancements, challenges remain in translating circRNA research into clinical practice. While the synthesis highlights promising findings, several limitations such as methodological variability, lack of standardization, and limited clinical validation must be addressed to fully translate circRNA research into clinical applications. The standardization of circRNA detection methods, large-scale validation studies, and the optimization of RNA-based therapeutics are essential for their successful clinical implementation. Interdisciplinary collaboration among oncologists, geneticists, bioinformaticians, and pharmaceutical researchers will be crucial in addressing these challenges and unlocking the full potential of circRNAs in cancer treatment.

circRNAs represent a promising frontier in precision medicine, offering new solutions for cancer diagnosis, prognosis, and therapy. As research continues to evolve, integrating circRNA profiling with pharmacogenomic approaches will be instrumental in refining treatment paradigms, overcoming drug resistance, and ultimately improving patient outcomes. With continued advancements, circRNA-based strategies have the potential to transform oncology, leading to more effective, individualized, and targeted therapeutic interventions.

## Figures and Tables

**Table 1:** Comparison of circRNA databases relevant to drug response.

Database	Focus on Drug Response	Strengths	Limitations	Reference *
circBase	Does not directly focus on drug response but offers foundational circRNA annotations	Serves as a foundational resource consolidating circRNA data from multiple studies.	Lacks pharmacogenomic-specific annotations, limiting its utility for drug interaction studies.	[125,126]
ncRNADrug	Provides insights into ncRNA roles in drug resistance mechanisms	Catalogs both validated and predicted interactions between ncRNAs (including circRNAs) and drug resistance.	Computational predictions may introduce biases without experimental validation.	[128]
CircNet 2.0	Facilitates study of circRNA-miRNA-mRNA interactions, relevant for drug response	Provides an interactive platform for circRNA regulatory networks, including circRNA-miRNA-mRNA interactions.	Does not explicitly focus on pharmacogenomics.	[129]
CircAtlas	Not specifically focused on drug response but may aid in exploring circRNA-drug interactions.	Provides detailed human circRNA data, including sequences, annotations and the potential circRNA–miRNA interactions	Not explicitly designed for drug response studies.	[130,131]
circRNADb	Provides annotations that can be used for exploring drug response mechanisms	Provides a comprehensive catalog of human exonic circRNAs with annotations.	Not specifically tailored for drug resistance mechanisms.	[127,131]
CircRiC	Cancer-specific circRNAs	Specializes in circRNA expression and drug sensitivity in cancer cell lines.	Focuses on cancer models, limiting its generalizability.	[132,133]
GATECDA Framework	Predicts circRNA influence on drug sensitivity	Leverages graph attention auto-encoder techniques to predict circRNA influence on drug sensitivity.	Computational framework may lack broad experimental validation.	[134]

* References [125,126,127,128,129,130,131,132,133,134] correspond to citation numbers in the main manuscript.

## Data Availability

Not applicable.
